# Ileocolonic Healing after Small Ileocecal Resection in Mice: NOD2 Deficiency Impairs Anastomotic Healing by Local Mechanisms

**DOI:** 10.3390/jcm12103601

**Published:** 2023-05-22

**Authors:** Maria B. Witte, Johannes Saupe, Johannes Reiner, Karen Bannert, Clemens Schafmayer, Georg Lamprecht, Peggy Berlin

**Affiliations:** 1 Department of General, Visceral, Thoracic, Vascular and Transplant Surgery, Rostock University Medical Center, Schillingallee 35, 18057 Rostock, Germany; 2 Division of Gastroenterology and Endocrinology, Department of Medicine II, Rostock University Medical Center, Ernst-Heydemann-Strasse 6, 18057 Rostock, Germany

**Keywords:** NOD2, ileocecal healing, anastomosis, muramy-dipeptide, matrix, matrix metalloproteinase, microbiome, Crohn’s disease

## Abstract

Ileocecal resection (ICR) is frequently performed in Crohn’s disease (CD). *NOD2* mutations are risk factors for CD. *Nod2* knockout (ko) mice show impaired anastomotic healing after extended ICR. We further investigated the role of NOD2 after limited ICR. C57B16/J (wt) and *Nod2* ko littermates underwent limited ICR including 1–2 cm terminal ileum and were randomly assigned to vehicle or MDP treatment. Bursting pressure was measured on POD 5, and the anastomosis was analyzed for matrix turn-over and granulation tissue. Wound fibroblasts from subcutaneously implanted sponges were used for comparison. The M1/M2 macrophage plasma cytokines were analyzed. Mortality was not different between groups. Bursting pressure was significantly decreased in ko mice. This was associated with less granulation tissue but was not affected by MDP. However, anastomotic leak (AL) rate tended to be lower in MDP-treated ko mice (29% vs. 11%, *p* = 0.07). mRNA expression of *collagen-1α (col1 α)*, *collagen-3α (col3 α)*, *matrix metalloproteinase* (*mmp*)*2* and *mmp9* was increased in ko mice, indicating increased matrix turn-over, specifically in the anastomosis. Systemic TNF-α expression was significantly lower in ko mice. Ileocolonic healing is impaired in *Nod2* ko mice after limited ICR by local mechanisms maybe including local dysbiosis.

## 1. Introduction

Ileocolonic anastomosis is a frequently performed operation for malignant or benign diseases. Failure of healing manifested as anastomotic leakage (AL) is the most feared postoperative complication resulting in prolonged hospital stay, increased costs and even death [1]. In Crohn’s disease (CD), ileocecal resection (ICR) is the most often performed resectional surgery. The risk of AL after this procedure is 3–5% in expert centers [2].

Ileocolonic healing has to proceed in the presence of bacteria on the luminal aspect of the anastomosis. The role of the microbiome in anastomotic healing has long been recognized but has recently received new interest [3,4] and has reached clinical attention in terms of preoperative oral decontamination in colorectal surgery in humans [5]. There are controversial data on whether the type of resection influences the postoperative microbiome. We have previously shown that resection of the cecum results in important changes in the stool microbiome in mice shifting the phyla toward an increase in Firmicutes and Proteobacteria [6,7]. Enterococcus belongs to Firmicutes and is capable of increasing local matrix metalloproteinase 9 (*mmp9*) activity, thereby possibly weakening anastomotic healing [8]. Experimental colonic healing is improved by *mmp* inhibitors [9,10]. Inhibition of NF-kB results in lower *mmp2* activity [11], an effect that might be mediated via TNF-α [12].

CD is associated with genetic risk profiles such as *NOD2* mutations [13,14]. The role of *NOD2* genetic variants for impaired ileocecal healing is unclear. In humans, studies have shown either no influence [15] or a negative association [16]. In addition, there are different *NOD2* genetic variants associated with CD, making studies in humans difficult to interpret. We have previously shown that *Nod2*-deficient mice (*Nod2* ko) have impaired ileocolonic healing measured by a lower bursting pressure and a higher collagen degrading anastomotic milieu [17]. NOD2 is a pattern recognition receptor (PPR) which recognizes intracellular bacterial fragments leading to activation of the NF-kB-mediated signaling. Muramyl–dipeptide (MDP) is the ligand of the NOD2 receptor [18]. Prior to the discovery of NOD2, MDP was used as a non-specific immunomodulator that improved survival in experimental peritonitis models [19]. In an excisional rodent wound model, *Nod2* deficiency delays healing [20]. Intraperitoneal administration of MDP results in activation of the NOD2 downstream signaling but also delays wound healing by reducing epithelialization [21]. Furthermore, MDP stimulates colonic cell proliferation in vitro [22]. This suggests that NOD2 and MDP play a central role in healing.

We have previously studied ileocolonic anastomotic healing and the influence of NOD2 deficiency after extensive ileocecal resection and thus under conditions of intestinal insufficiency and adaptation. Of note, in this model, most of the Paneth cell mass as an important NOD2 effector is removed and is not restored at post-operative day 5 when anastomotic healing is assessed. Importantly, significant dysbiosis occurs even after limited ileocecal resection. Here, we address the role of NOD2 deficiency on anastomotic healing more directly, omitting the influence of intestinal insufficiency and the loss of Paneth cell mass and reflecting a common surgical scenario.

## 2. Material and Methods

### 2.1. Animal Monitoring

All animal experiments were performed according to the EU Directive 2010/63/EU of the animal protection law and approved by the local animal committee (Landesamt für Landwirtschaft, Lebensmittelsicherheit und Fischerei, Mecklenburg-Vorpommern, 722.3-1.1-067/19).

Animal breeding and housing was performed as previously described [23]. Briefly, male C57BL6/J (wt) mice and B6.129S1-Nod2^tm1Flv^/J (*Nod2* ko) mice on a C57BL6/J background were purchased from Jackson Laboratory (Bar Harbor, ME, USA) and bred in the animal facilities at the Institute for Experimental Surgery, University Medical Center Rostock. Animals were allowed to acclimatize and had free access to chow and water. Two days before surgery and until the end of the experiment, mice were switched to liquid chow (AIN 93G, ssniff, Soest, Germany). Animals were weighed and examined for wellbeing daily in the morning [24]. If mice had a wellness score below 4 or lost more than 30% of their initial weight, they were killed and autopsied. Stool water content was measured on the day of surgery and on days 2 and 5 afterwards as described previously [23]. In brief, fresh stool was collected in pre-weighed tubes [e], which were immediately closed. After weighing again (w), the tubes with fresh stool were opened and placed in a hot air oven at 85 °C. After 24 h, the tubes were once again weighed (d) and stool water content (%) was calculated as follows: (w − d)/(w − e) × 100.

### 2.2. Operative Procedure

Mice were anesthetized using ketamine/xylazine intraperitoneally. Median laparotomy and eventration of the distal small bowel and cecum were performed. Approximately 1–2 cm of the terminal ileum and the cecum were resected. Mesenteric vessels were clipped using microclips (Weck, Horizon™, Teleflex Medical, Fellbach, Germany). The ascending branch of the ileocolic vessels and the arcade to the ileum were conserved to assure optimal perfusion. The ileocolonic anastomosis was performed with single interrupted stiches using 10-0 monofilament suture material under an operating microscope with a 16× magnification. Adhesion prophylaxis was performed with a 1 mL saline irrigation before closure of the abdomen. The fascia was closed with a 6-0 prolene suture. Two subcutaneous pockets were created on both sides of the fascial closure and one PVA sponge was placed into each one. The skin was closed with interrupted sutures. Mice were resuscitated with a 1 mL NaCl 0.9% solution and received a 5 mg/kg bw carprofen as analgesic, both subcutaneously. They were kept in a warming terrarium for at least 4 h before returning to the cages.

In total, 43 male adult wildtype C57BL6/J mice (wt) and 45 adult *Nod2* knockout mice (ko) at the age of 3 to 6 months were randomized into four groups and received either muramyl dipeptide (MDP, 100 µg/mouse, Bachem, Bubendorf, Switzerland) or vehicle (veh, saline) in a volume of 100 µL intraperitoneally, two hours prior to surgery, and again two and five days after surgery. Mice were killed 3 h after the last injection.

### 2.3. Bursting Pressure Measurement

Mice were anesthetized with ketamine/xylazine on postoperative day 5. A relaparotomy was performed and the anastomosis was visualized without perturbing the adhesions. An infusion catheter was introduced 2 cm proximal to the anastomosis and a transducer catheter 2 cm distal to the anastomosis. Both segments were then ligated with nylon sutures. Saline was injected at 1 ml/min using an infusion pump (Lineomat, VEB MLW, Berlin, Germany). The increasing pressure was measured continuously and a sudden drop in pressure and extravasation of fluid from the anastomosis indicated bursting. The maximum pressure before the drop was recorded as the bursting pressure. The animals were euthanized at the end of the measurement by cervical dislocation.

### 2.4. Tissue Harvest

The anastomosis including bowel segments 5 mm proximal and 5 mm distal was harvested for analysis. The tissue was longitudinally cut in half, and either fixed in 4% formalin or snap-frozen in liquid nitrogen. Frozen tissues were stored at −80° for subsequent RNA or protein analyses. The tissue used for bursting pressure measurement was not used for RNA isolation.

### 2.5. Protein Isolation and Hydroxyproline (OHP) Measurement

For OHP measurement, 10 mg of tissue from the snap-frozen anastomosis was shredded and hydrolyzed in 37% HCl for 3 h at 120 °C. After centrifugation, the supernatant was analyzed by chloramine T using the Hydroxyproline Assay Kit MAK008 from Sigma-Aldrich (Steinheim, Germany). Then, 20 µL lysate was added into a 96-well plate. Standards were run in parallel. All samples were run in duplicate following the manufacturers protocol. Analysis was performed using the Glomax Multi Detection System at 560 nm absorption. Results were normalized for protein content which was determined by the Bradford method.

### 2.6. RNA Isolation

RNA isolation was performed using the RNeasy Minikit (Qiagen, Hilden, Germany). Briefly, 30 mg tissue was homogenized using a tissue lyser at 50 Hz for 5 min. After centrifugation at 14.000× *g* for 3 min, the supernatant was mixed with an equal volume 70% ethanol and then applied on an RNeasy column following the manufacturer’s instructions. DNA digestion at room temperature was performed using the RNase-free DNase set (Qiagen, Hilden, Germany). RNA quality was checked by agarose gel electrophoresis. cDNA synthesis using 2 µg RNA as template was performed using the high capacity cDNA reverse transcription kit (Applied Biosystems, Darmstadt, Germany).

### 2.7. Quantitative Real-Time PCR

Gene expression was analyzed by quantitative real-time PCR using the TaqMan™ Universal PCR Master Mix and the pre-designed TaqMan^®^ gene expression assays (Supplemental Table S1). In total, 15 ng of cDNA was used per PCR reaction. Analyses were performed in triplicate on a ViiA7 sequence detection system (Applied Biosystems, Thermo Fisher Scientific, Karlsruhe, Germany). PCR conditions were as follows: 95 °C for 10 min, 55 cycles of 15 s at 95 °C, 1 min at 60 °C. The data were normalized to GAPDH. Expression levels of the genes of interest are provided as 2^−ΔcT^.

### 2.8. Histology

The excised and formalin-fixed tissue of the anastomosis was further processed to obtain longitudinally oriented tissue samples which were paraffin-embedded and cut into 4 µm sections using an electronic rotary microtome (Microm HM340E, Thermo Scientific^TM^, Karlsruhe, Germany). The tissue slices were stained with hematoxylin and eosin (HE). Technically well-processed tissue sections of the anastomoses were analyzed by two different individuals in a blinded fashion using an Axio Observer inverted microscope (Zeiss, Oberkochen, Germany) at 10× magnification and the Zen 2.3 software.

### 2.9. Naphtol-AS-D-Chloresterase (ASD) Staining

Paraffin-embedded tissue slices were subjected to the Naphtol-AS-D-chloresterase (ASD) reaction to visualize granulocytes. A total of 10 mg of diazonium dye (fast garnet GBC salt, Sigma-Aldrich, Taufkirchen, Germany) was dissolved in 100 mL 5% *v*/*v* phosphate buffered saline (stock solution: 0.16 M Na_2_HPO_4_ * 2H_2_O, 0.04 M KH_2_PO_4_, 2.74 M NaCl). A total of 16 mg of Naphtol AS-D chloracetate (PanReac AppliChem, Darmstadt, Germany) was dissolved in 2 mL of DMSO. Dissolved diazonium dye and Naphtol AS-D chloracetate solution were gently mixed and the staining solution was filtered. The tissue slices were deparaffinized and incubated in filtered ASD staining solution for 1 h at room temperature. The slices were washed in distilled water and counterstained with hemalaun (Carl Roth, Karlsruhe, Germany) for 5 min. The slices were air-dried and embedded in a 37 °C prewarmed Kaiser’s glycerol gelatine (Sigma Aldrich, Taufkirchen, Germany). Anastomoses were imaged with a Zeiss cell observer microscope at a 20× magnification. Positive stained cells and the area of granulation tissue were calculated using the open source QuPath 0.4.3 software.

### 2.10. Quantification of Anastomotic Granulation Tissue

The amount of anastomotic granulation tissue was quantified by HE staining using the area measurement function of the QuPath software 0.4.3. The anastomotic area was calculated in µm². The number of ASD-positive cells within this area was counted. The number of cells was divided by the area. Quantification was performed in a blinded manner by two different observers.

### 2.11. Isolation of Wound Fibroblasts

Four sterilized and moisturized PVA sponges (IVALON^®^ PVA sponge M-PACT, Eudora, KS, USA) cut to a 6 mm diameter size were implanted subcutaneously after closure of the fascia by creating two small pockets on each side. They were extracted under sterile conditions and transferred to Dulbecco’s modified Eagles media (DMEM, Gibco^TM^ Fisher Scientific, Karlsruhe, Germany) supplemented with GlutaMax™ (Gibco^TM^, Fisher Scientific, Karlsruhe, Germany), 10% *v*/*v* fetal calf serum (Pan-Biotech, Aidenbach, Germany) and 0.5% *v*/*v* penicillin/streptomycin (Gibco^TM^ Fisher Scientific, Karlsruhe, Germany). The sponges were maintained overnight in an incubator (37 °C, 5% CO_2_). The cells were removed from the sponges by repetitive squeezing and flushing in media the next day. The isolated cells were washed three times and seeded with complete media in 75 cm^2^ cell culture flasks (Sarstedt, Nümbrecht, Germany) for 7 to 10 days. Confluent fibroblasts were passaged by trypsinization (0.25% trypsin-EDTA, Gibco, Karlsruhe, Germany). Fibroblasts (0.5 × 10^6^ total cells) between passage 1 and 4 were used for RNA isolation using the RNeasy Micro Kit (Qiagen, Hilden, Germany) according to the manufacturer’s protocol. Sponges from different mice of the same group were pooled to obtain a higher cell yield.

### 2.12. Multiplex Analysis of Plasma Cytokines

M1/M2 macrophage cytokines (KC, TGF-ß1, IL-18, IL-23, CCL22, IL-10, IL-12p70, IL-6, TNF-α, G-CSF, CCL17, IL-12p40, IL-1ß) in the plasma of mice were measured using the multi-analyte bead-based flow assay kit LEGENDplex^TM^ (No. 740845, Biolegend^®^, Koblenz, Germany) according to the manufacturer’s instructions. In brief, retrobulbary obtained blood was collected in EDTA tubes at the day of harvest and centrifuged at 1000× *g* for 20 min. Plasma was stored at −20 °C until use. Thawed samples were vortexed and centrifuged again to remove particulates. Pre-mixed beads were vortexed and all kit reagents were prepared at room temperature as instructed. Serial 1:4 dilutions of the provided panel standard were performed and plasma samples were diluted 2-fold with an assay buffer. Standards and samples including non-operated wt and ko mice as basal values were loaded on a 96-well plate in duplicates. Mixed beads were added to each well and the sealed plate was incubated in the dark on a plate shaker at 600 rpm for 2 h. The plate was washed twice, and a biotinylated detection antibody was added to each well for 1 h at 600 rpm in the dark. Streptavidin–phycoerythrin was added without washing and incubated for 30 min in the dark at 600 rpm. The plate was washed twice and read directly on a flow cytometer with autosampler. A total of 3.900 beads was acquired and samples were read at low flow rate. The LEGENDplex^TM^ cloud-based data analysis software (legendplex.qognit.com, version 2021-04-15) was used for the analysis of FCS files.

### 2.13. Statistics

For the graphs and statistics of stool water, body weight, wellness score, and food intake, only data from mice that survived the expected end point were considered. Mice that had to be sacrificed earlier were excluded.

Numerical data were first tested for Gaussian distribution, then either analyzed by ANOVA or unpaired Student’s *t*-test or by Kruskal–Wallis and Mann–Whitney U test considering *p* < 0.05 as significance level using GraphPad Prism, (GraphPad Software, version 8.4.3, San Diego, CA, USA). Results are displayed as mean with SD as indicated in the legends of the figures.

## 3. Results

### 3.1. Wellness Score, Vital Parameters and Survival

**Figure 1:**
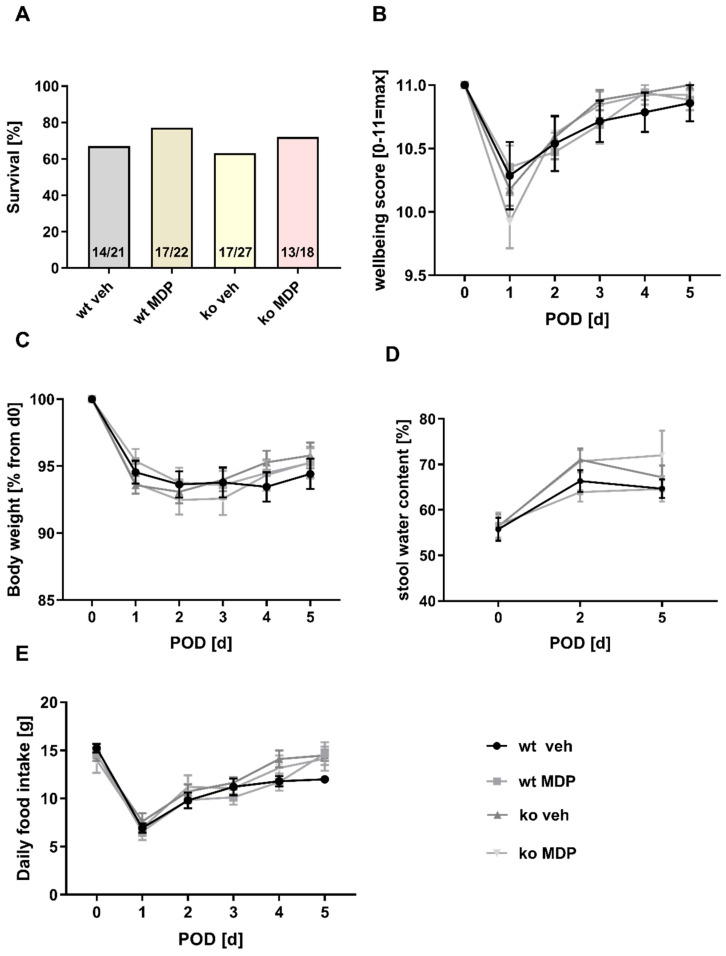
Survival (**A**), wellbeing (**B**), body weight (**C**), stool water content (**D**), and food intake (**E**) of wildtype (wt) and *Nod2* knockout (ko) mice with limited ileocecal resection and ileocolonic anastomoses. Mice received intraperitoneal injections of muramyl dipeptide (MDP, 100 µg/mouse) or NaCl (vehicle, veh 100 µL) 2 h prior to surgery, at day 2 and day 5. (**B**–**E**): Only data from mice that survived the expected end point were considered. Mice that had to be sacrificed earlier were excluded. Values are given as mean ± SD, (**B**): Wildtype treated with vehicle (wt veh) n = 14; wildtype treated with muramyl dipeptide (wt MDP) n = 17; *Nod2* knockout treated with vehicle (ko veh) n = 17; Nod2 knockout treated with muramyl dipeptide (ko MDP) n = 13; no differences between the groups (*p* < 0.05; 2-way ANOVA), (**C**): wt veh n = 14; wt MDP n = 17; ko veh n = 17; ko MDP n = 13; no differences between the groups (Kruskal–Wallis test *p* < 0.05), (**D**): wt veh n = 10; wt MDP n = 10; ko veh n = 13; ko MDP n = 10; no differences between the groups (*p* < 0.05; 2-way ANOVA). (**E**): wt veh n = 14; wt MDP n = 17; ko veh n = 18; ko MDP n = 13; no differences between the groups (*p* < 0.05; 2-way ANOVA).

There was no phenotypic difference between wt and ko mice. Survival after surgery was numerically higher in wt and MDP-treated mice in general. However, this was not statistically significant (Figure 1A). Wellness score, body weight, food intake and stool water content were used for animal monitoring (Figure 1B–E). There was neither a significant difference between wt and ko mice nor did MDP treatment affect these parameters. The difference in survival between wt and ko mice was therefore not due to a difference in wellbeing.

### 3.2. Anastomotic Healing Measured by Leak Rate, Bursting Pressure and Formation of Granulation Tissue

**Figure 2:**
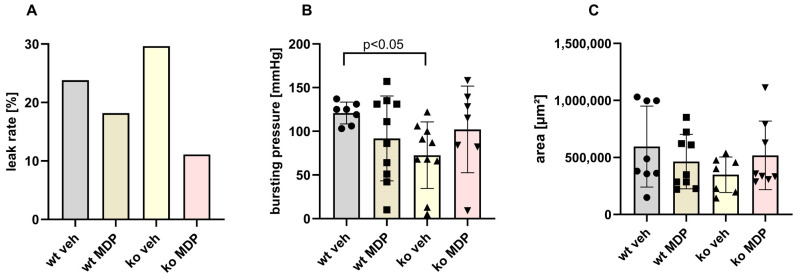
Anastomotic leak rate (**A**), bursting pressure (**B**), and granulation tissue area (**C**) of ileo-colonic anastomosis from wildtype (wt) and *Nod2* knockout (ko) mice at day 5 after ileocecal resection. Mice received intraperitoneal injections of muramyl dipeptide (MDP, 100 µg/mouse) or NaCl (vehicle, veh 100 µL) 2 h prior to surgery, at day 2 and day 5, 2 h before harvest. (**A**), leak rate *p* = 0.07 by Student’s *t*-test between ko veh and ko MDP. B, C: values are given as mean ± SD, (**B**): Wildtype treated with vehicle (wt veh) n = 7; wildtype treated with muramyl dipeptide (wt MDP) n = 10; *Nod2* knockout treated with vehicle (ko veh) n = 10; Nod2 knockout treated with muramyl dipeptide (ko MDP) n = 7, *p* = 0.0064 wt veh vs. ko veh; unpaired *t*-test. (**C**): wt veh n = 8; wt MDP n = 9; ko veh n = 7; ko MDP n = 8, No differences between the groups (*p* < 0.05; 2-way ANOVA).

Overall, AL occurred in 23% of wt vs. 29% of ko mice and was the main cause of death in all groups. MDP treatment lowered the leak rate in both genotypes and predominantly in ko mice (29% in ko veh vs. 11% in ko MDP, Figure 2A).

Bursting pressure was significantly lower in ko mice compared to wt mice (Figure 2B). MDP treatment did not affect bursting pressure neither in wt nor ko mice.

The OHP concentration in the anastomosis was significantly higher in ko veh mice compared to wt veh mice (11.09 ± 0.85 vs. 8.50 ± 0.62 mg/mg protein) but unaffected by MDP treatment.

**Figure 3:**
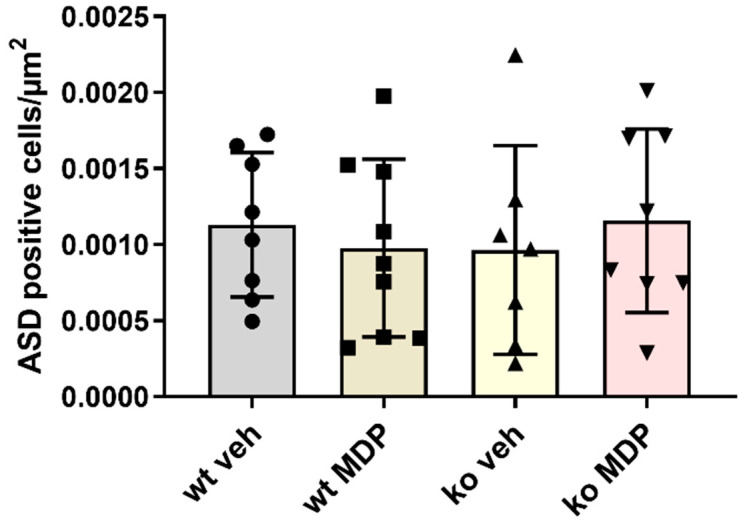
Number of Naphtol-AS-D-chloresterase (ASD)-positive stained cells per area of granulation tissue (cells/µm^2^) in the anastomoses of wildtype (wt) and *Nod2* knockout (ko) mice 5 days after ileocecal resection. Mice received intraperitoneal injections of muramyl dipeptide (MDP, 100 µg/mouse) or NaCl (vehicle, veh 100 µL) 2 h prior to surgery, at Day 2 and Day 5. Values are given as mean ± SD. Wildtype treated with vehicle (wt veh) n = 8; wildtype treated with muramyl dipeptide (wt MDP) n= 9; *Nod2* knockout treated with vehicle (ko veh) n = 7; Nod2 knockout treated with muramyl dipeptide (ko MDP) n = 8. No differences between the groups (Kruskal–Wallis test).

Sections perpendicular to the anastomosis were used to quantify the formation of the granulation tissue. The area of the newly formed granulation tissue was not statistically different between the groups (Figure 2C). The number of granulocytes expressed as ASD-positive cells per newly formed tissue was also not different between the groups (Figure 3). Interestingly, there was a strong correlation between the number of ASD-positive cells and the tissue area suggesting “regular coordinated” healing in wt veh mice. This correlation was present neither in ko veh mice nor in wt MDP mice but restored in ko MDP mice (Supplemental Figure S1).

### 3.3. mRNA Expression of Proteins Related to Matrix Turnover in Anastomosis

**Figure 4:**
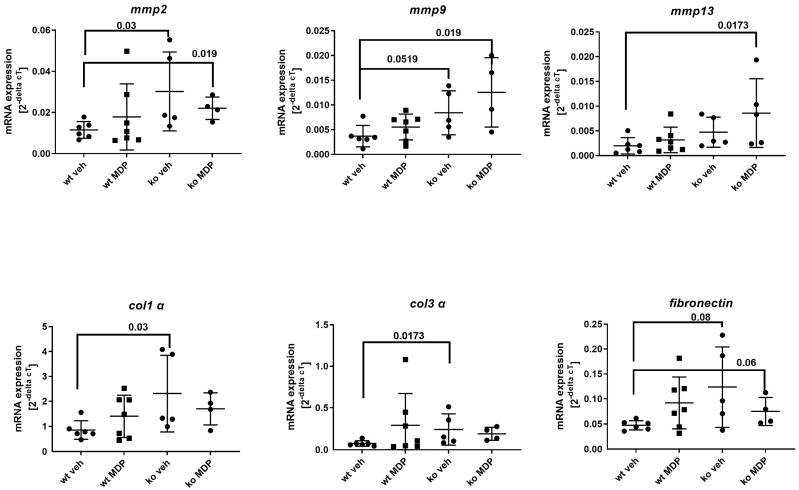
mRNA gene expression of the matrix proteins *matrix metalloproteinase* (*mmp*) *2*, *9*, and *13* as well as *collagen 1 alpha 1* (*col1 alpha*) and *collagen 3 alpha 1* (*col3 alpha*) and *fibronectin* in the anastomoses of wildtype (wt) and *Nod2* knockout (ko) mice 5 days after ileocecal resection. Mice received intraperitoneal injections of muramyl dipeptide (MDP, 100 µg/mouse) or NaCl (vehicle, veh 100 µL) 2 h prior to surgery, at day 2 and day 5. Values are given as mean ± SD. Wildtype treated with vehicle (wt veh) n = 6; wildtype treated with muramyl dipeptide (wt MDP) n= 7; *Nod2* knockout treated with vehicle (ko veh) n = 5; *Nod2* knockout treated with muramyl dipeptide (ko MDP) n = 4. *p* values as indicated in the figure. Mann–Whitney U test.

The mRNA expression of *collagen 1 alpha* (*col1 α*), *collagen 3 alpha* (*col3 α*) and *fibronectin* was analyzed as a parameter for matrix synthesis in anastomotic tissue. ko veh mice displayed significantly elevated mRNA for these matrix proteins (Figure 4, lower panel), an effect that was not influenced by MDP. Transcripts of matrix-degrading enzymes *mmp2* and *mmp9* were also significantly elevated, but not those of *mmp13* (Figure 4, upper panel). The higher OHP concentration seen in ko veh mice reflects the higher matrix breakdown products. In summary, this suggests that an increased matrix turnover takes place at the anastomosis.

### 3.4. mRNA Expression of Proteins Related to Matrix Turnover in Wound-Derived Fibroblasts

**Figure 5:**
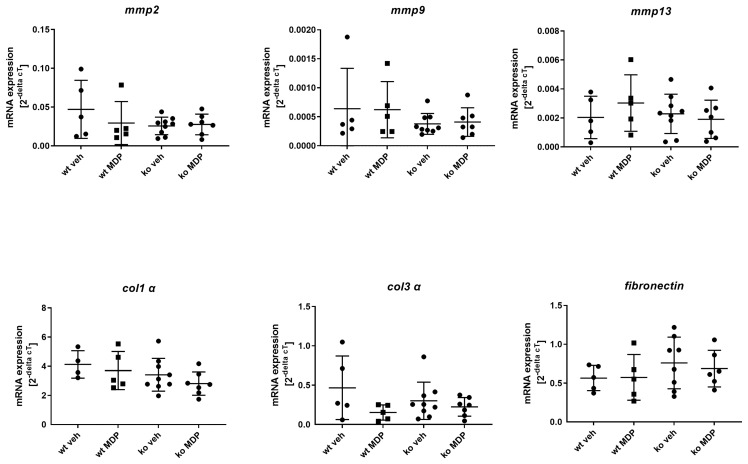
mRNA gene expression of the matrix proteins *matrix metalloproteinase* (*mmp*) *2*, *9*, and *13* as well as *collagen 1 alpha* (*col1 α*) and *collagen 3 alpha* (*col3 α*) and *fibronectin* in wound cell fibroblasts which were isolated at day 5 after ileocecal resection from wildtype (wt) and *Nod2* knockout (ko). Mice received intraperitoneal injections of muramyl dipeptide (MDP, 100 µg/mouse) or NaCl (vehicle, veh 100 µL) 2 h prior to surgery at day 2 and day 5. Fibroblast were cultured in vitro and used at passage 1–3 for RNA isolation. Values are given as mean ± SD. Wildtype treated with vehicle (wt veh) n = 4–5; wildtype treated with muramyl dipeptide (wt MDP) n= 5; *Nod2* knockout treated with vehicle (ko veh) n = 8–9; *Nod2* knockout treated with muramyl dipeptide (ko MDP) n = 6–7. No differences between the groups (Kruskal–Wallis test).

mRNA expression of *col1 α*, *col3 α* and *fibronectin* displayed no significant differences between wt and ko mice at postoperative day 5 and were not affected by MDP treatment (Figure 5, lower panel). Transcripts of matrix degrading enzymes *mmp2*, *mmp9* and *mmp13* were also not affected by genotype or MDP treatment (Figure 5, upper panel).

### 3.5. Cytokine Expression in the Anastomosis and in Wound Derived Fibroblasts

**Figure 6:**
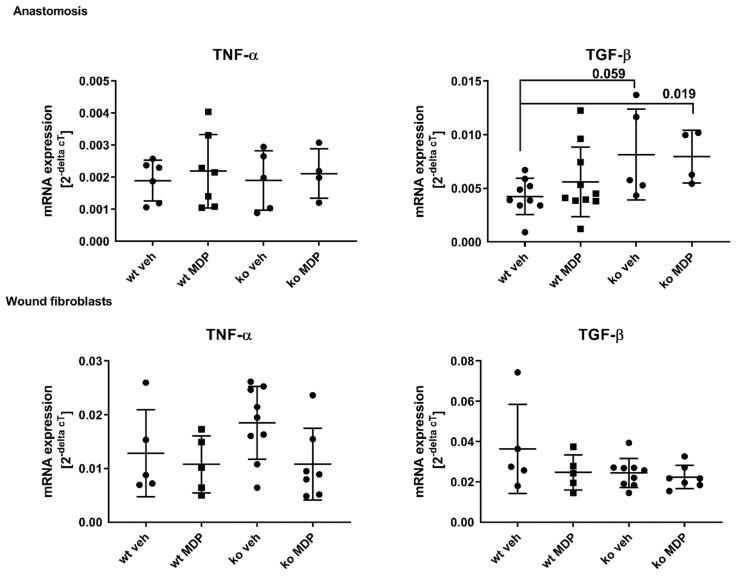
Tumor necrosis factor-α (TNF-α), interleukin 12 (IL12p40), C-C motif chemokine ligand (CCL) 17, and 22 concentration in the plasma of wildtype (wt) and *Nod2* knockout (ko) mice at day 5 after ileocecal resection. Mice received intraperitoneal injections of muramyl dipeptide (MDP, 100 µg/mouse) or NaCl (vehicle, veh 100 µL) 2 h prior to surgery at Day 2 and Day 5. Values are given as mean ± SD. Wildtype treated with vehicle (wt veh) n = 9–10; wildtype treated with muramyl dipeptide (wt MDP) n = 9–10; *Nod2* knockout treated with vehicle (ko veh) n = 5–8; *Nod2* knockout treated with muramyl dipeptide (ko MDP) n = 6–10. *p* values as indicated in the figure. Mann–Whitney U test.

TGF-ß and TNF-α were assessed as key cytokines for wound healing. TGF-ß regulates collagen synthesis and TNF-α is the down-stream cytokine of NF-kB-mediated NOD2 signaling. TGF-ß mRNA expression was significantly higher in the anastomosis of ko veh and ko MDP mice compared to wt mice (Figure 6, left upper panel). TNF-α mRNA expression, however, was not different (Figure 6, right upper panel).

TNF-α and TGF-ß mRNA expressions in WFB were equal between groups (Figure 6, lower panels).

### 3.6. Plasma Cytokines

**Figure 7:**
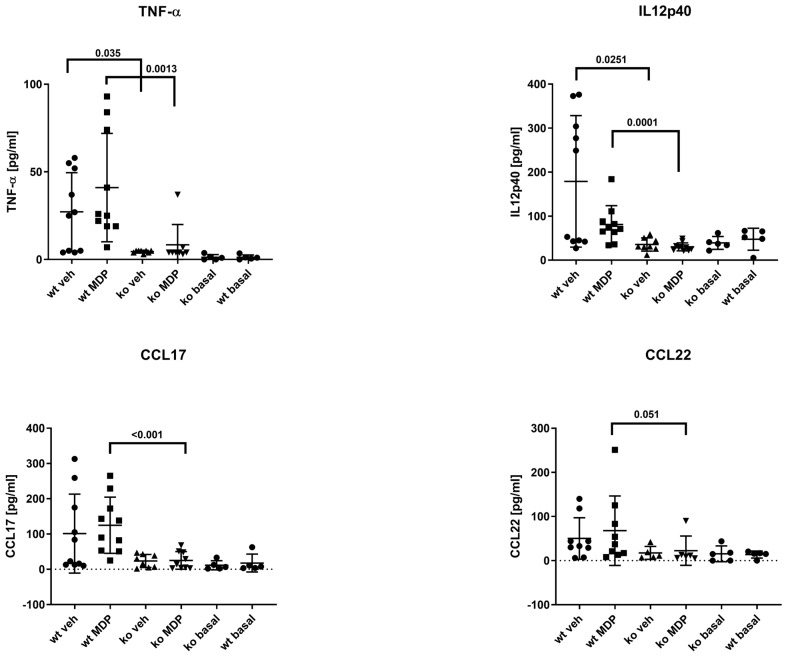
mRNA gene expression of *Tumor necrosis factor-α* (*TNF-α*) and *Transforming growth factor-ß* (*TGF-ß*) in the anastomoses (upper lane) or in wound fibroblast (lower lane) derived from wildtype (wt) and *Nod2* knockout (ko) mice at Day 5 after ileocecal resection. Mice received intraperitoneal injections of muramyl dipeptide (MDP, 100 µg/mouse) or NaCl (vehicle, veh 100 µL) 2 h prior to surgery at Day 2 and Day 5. Values are given as mean ± SD. Wildtype treated with vehicle (wt veh) n = 5 and 7 (*TNF-α*) or n = 5 and 9 (*TGF-ß*); wildtype treated with muramyl dipeptide (wt MDP) n = 5 (*TNF-α*) or n = 5 and 10 (*TGF-ß*); *Nod2* knockout treated with vehicle (ko veh) n = 9 (*TNF-α*) or n = 5 and 9 (*TGF-ß*); *Nod2* knockout treated with muramyl dipeptide (ko MDP) n = 4 and 7 (*TNF-α*) or n = 4–7 (*TGF-ß*); *p* values as indicated in the figure. Mann–Whitney U test.

Macrophage-derived plasma cytokines were investigated to monitor the systemic inflammatory status of mice after resection. Plasma from non-operated mice served as basal controls (wt basal and ko basal). There were no differences for inflammatory M1/M2-derived cytokines on postoperative day 5 for IL-1, IL-6, IL-10, IL-18, IL-23, and TGF-ß between wt and ko mice (not shown). There were, however, significantly lower plasma levels of TNF-α and IL12p40 in ko mice compared to wt mice (Figure 7). Additionally, MDP-treated ko mice had significantly lower CCL 17 and CCL 22 levels compared to MDP-treated wt mice.

## 4. Discussion

The present data show that ileocolonic healing after limited ileocecal resection removing 2 cm of the terminal ileum is impaired in *Nod2* ko mice compared to wt mice but unaffected by the NOD2 ligand MDP. The effect is predominantly local at the anastomosis since systemic parameters including macrophage-derived cytokine plasma levels as well as functional parameters from wound fibroblast on postoperative day 5 are unaffected by the *Nod2* status.

Anastomotic healing was assessed by leak rate, bursting pressure, formation of granulation tissue and matrix turn-over. The main parameters affected in *Nod2* ko mice were the bursting pressure and the mRNA expression of proteins related to matrix turn-over (*col1 α*, *col3 α* and *mmp2* and *mmp9*). Bursting pressure was significantly lower in *Nod2* ko mice than in the wt mice. At the same time, we detected only a trend towards a higher leak rate. This is most likely due to the formation of adhesions which were left in place in our experiments and which are an important compensatory mechanism in rodents preventing gross leakage. In line with this, adhesions are judged as a negative indicator of healing in a recently developed anastomotic healing score [25]. We chose bursting pressure as the quantitative functional parameter for anastomotic healing because it better reflects healing by matrix synthesis.

Bursting pressure may be reflected by the amount of granulation tissue. Similar to our previous study, neither the amount of granulation tissue nor the number of granulocytes per tissue area were different between wt and ko mice [17]. Interestingly, the strong positive correlation between the number of granulocytes per area was lost in ko veh mice indicating that healing was disturbed. This is in line with our previous finding that Nod2 ko mice do not display healing in the canonical manner. It may be speculated that the restauration of this deficit by MDP treatment in ko mice as shown in Supplemental Figure S1 is reflected by the lower AL seen in ko MDP-treated mice (Figure 2A). This would suggest that the effect of MDP is not NOD2-mediated.

### 4.1. The Effect of NOD2 Deficiency on Ileocolonic Anastomotic Healing Is Local

Wound-derived fibroblasts (WFB) isolated from subcutaneously implanted PVA sponges reflect healing distant from the anastomosis in a sterile, non-contaminated milieu. Since WFB change their phenotype with higher passages in vitro [26], they were used at an early stage. In sharp contrast to the situation at the anastomosis, mRNA expression of proteins involved in matrix turn-over was affected neither by *Nod2* deficiency nor by MDP treatment in wound-derived fibroblasts.

Macrophages are important contributors to the healing process. Up- or down-regulation of macrophage-derived cytokines influences the inflammatory phase. The majority of the M1/M2-derived cytokines were elevated after surgery in wt mice, indicating a general inflammatory response. In ko mice, however, the levels were lower in general, and significantly lower for G-CSF, TNF-α and IL12p40, reflecting specific alteration in the cytokine profile by NOD2.

### 4.2. Local Dysbiosis May Be Involved in Impaired Anastomotic Healing

MDP is the intracellular NOD2 ligand leading to upregulation of NOD2 and NF-kB mediated downstream signaling. NOD2 is predominantly expressed in the Paneth cells of the ileum [27], in antigen-presenting cells [28], but also in colonic crypts [22] and keratinocytes [21]. In the gastrointestinal tract, NOD2 regulates the host–microbial interaction [18] partially by defensin secretion [29]. NOD2 signaling therefore implicates a positive effect on healing. Since we have previously shown that complete removal of the Paneth cell pool by resection of a 12 cm terminal ileum in wt mice does not impair healing [17], the effect does not seem to be mediated directly through the Paneth cell mass and its NOD2-mediated defensin secretion.

*NOD2* mutations have been associated with increased permeability of the epithelium and decreased bacterial clearance, leading to higher susceptibility for CD in humans [30,31]. Resection of the cecum leads to dramatic decrease in α-diversity independent of the length of the resected small bowel, whereas resection of the small bowel alone does not [6,7,32]. Whether the microbiome of *Nod2* mice is different per se is controversial [7,33,34]. Resection-induced changes in the phyla were altered in *Nod2*-deficient mice [23], possibly leading to a specific local alteration. The role of Enterococci in CD is controversial; the NOD2-mediated effects of Enterococci may either protect or harm the host [35,36]. In this context, it must be kept in mind that models of colitis-implicating epithelial inflammation are not comparable with full-thickness injury of the bowel and subsequent anastomotic healing.

The role of the microbiome for successful healing has recently been underlined by fecal microbiota transfer (FMT) from humans with clinical AL to mice inducing AL in the experimental setting [37]. In this setting, *Alistipes onderdonkii*, a Gram-negative member of the phylum bacteroidota, has detrimental effects on colonic healing. In most experimental settings, changes in luminal phyla are judged representative. However, luminal and local phyla at the anastomotic site might be significantly different [6,38]. Of note, in our experimental setting, mice did not receive perioperative antibiotics not to interfere with “normal” changes in postoperative α-diversity [39,40]. The upregulation of *mmp2* and *mmp9* in *Nod2* ko mice could be a reflection of specific local bacterial *mmp* induction. Stimulation of PBMC by Gram-positive bacteria such as Enterococci or by MDP in vitro leads to a significant induction of *mmp9* activity, whereas Gram-negative bacteria fail to do so. This effect is abrogated in patients with CD and *NOD2* mutation [41]. The postoperative bacterial shift could therefore influence local *mmp* synthesis. *Enterococcus faecalis* has been implicated as a negative player for healing by increasing local *mmp2* and *9* activity, leading to weaker anastomosis [8,42]. In addition, early detection of *Enterococcus* in drain fluids has been related to upcoming AL [43].

On the other hand, some phyla may indirectly contribute positively to anastomotic healing by producing short-chain fatty acids (SCFA), which are the substrate for epithelial cell proliferation [44]. Furthermore, bacterial derived SCFAs also modulate *mmp* production [45].

Limitation of the study: It is estimated that homozygous or heterozygous *NOD2* mutations are prevalent in up to 40% of patients with Crohn’s disease [46,47]. In the experimental setting, there is a frameshift model resulting in a gain of function with overexpression of NFkB [48] and a loss of function model using *Nod2* knockout mice with complete *Nod2* deficiency [49]. In our model, we used the same *Nod2*-deficient mouse, resulting in a loss of a NOD2 receptor. However, the *Nod2*-deficient mice do not reflect the human genetic variants associated with Crohn’s disease [13,14], and the results shown herein might therefore not be directly transferrable into the clinical setting.

Adequate perfusion is mandatory for anastomotic healing. Although we conserved the vascular arcades for optimal perfusion at the bowel edges, we cannot rule out that differences in microperfusion exit between mice genotypes. ICG (indocyanine green) angiography has been established in colorectal surgery for assessing bowel perfusion, and its intraoperative application prevents AL [50]. However, ICG application is limited to minimally invasive surgery and has not yet been used in open ileocolonic healing in mice.

In summary, *Nod2*-deficient mice show impaired ileocolonic healing after limited ileocecal resection. The effect of *Nod2* deficiency occurs locally at the anastomosis, leading to weaker anastomosis due to modulation of *mmp2* and *mmp9* expression, which may be related to NOD2-dependent local dysbiosis.

## Data Availability

Data are available on request from the corresponding author.

## Supplementary Materials

The following supporting information can be downloaded at: https://www.mdpi.com/article/10.3390/jcm12103601/s1.
