# Supplementary material for: Ileocolonic Healing after Small Ileocecal Resection in Mice: NOD2 Deficiency Impairs Anastomotic Healing by Local Mechanisms
Source: J Clin Med. 2023 May 22;12(10):3601. doi: 10.3390/jcm12103601 (PMC10219437; doi:10.3390/jcm12103601)

Supplemental Table S1

Gen	Name	Assay-ID
MMP 2	Matrix metalloproteinase-2	Mm00439498_m1
MMP 9	Matrix metalloproteinase-9	Mm00442991_m1
MMP 13	Matrix metalloproteinase-13	Mm00439491_m1
Col1a1	Collagen 1 alpha 1	Mm00801666_g1
Col3a	Collagen 3 alpha	Mm01254476_m1
TNFα	Tumor necrosis factor alpha	Mm00443258_m1
TGFß	Transforming growth factor beta	Mm01178820_m1
GAPDH	Glyceraldehyde 3-phosphate dehydrogenase	Mm99999915_g1

Supplemental Figure S1

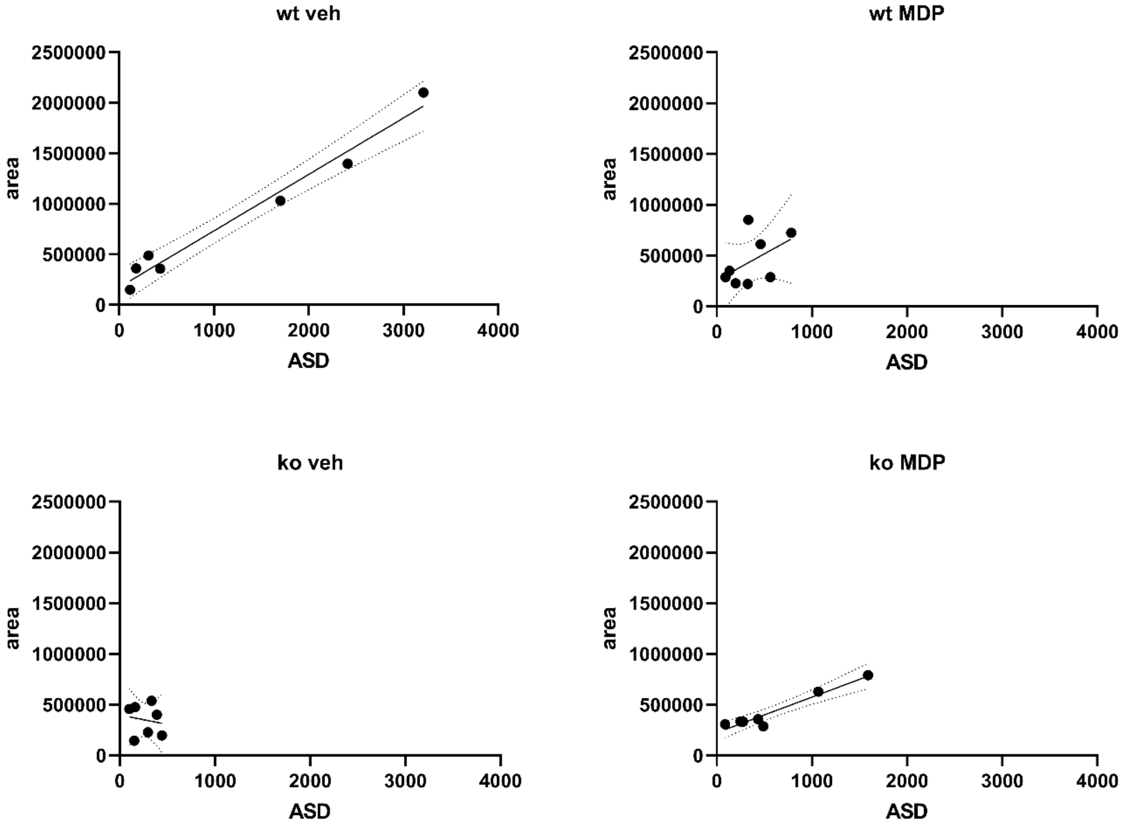

Supplement: Supplementary file 1 [file jcm-12-03601-s001.zip › jcm-2354765-supplementary.pdf]
